# Body-axis organization in tetrapods: a model-system to disentangle the developmental origins of convergent evolution in deep time

**DOI:** 10.1098/rsbl.2022.0047

**Published:** 2022-04-06

**Authors:** Borja Figueirido, Francisco J. Serrano, Alejandro Pérez-Ramos, Juan Miguel Esteban, Humberto G. Ferrón, Alberto Martín-Serra

**Affiliations:** ^1^ Departamento de Ecología y Geología, Facultad de Ciencias, Universidad de Málaga, 29071-Málaga, Spain; ^2^ Dinosaur Institute, Natural History Museum of Los Angeles County; Exposition Boulevard, Los Angeles, CA 90007, USA; ^3^ Instituto Cavanilles de Biodiversidad y Biologia Evolutiva, Universitat de Valencia, 46980-Paterna, Valencia, Spain

**Keywords:** macroevolution, development, phenotypic evolution, tetrapod axis

## Abstract

Convergent evolution is a central concept in evolutionary theory but the underlying mechanism has been largely debated since *On the Origin of Species*. Previous hypotheses predict that developmental constraints make some morphologies more likely to arise than others and natural selection discards those of the lowest fitness. However, the quantification of the role and strength of natural selection and developmental constraint in shaping convergent phenotypes on macroevolutionary timescales is challenging because the information regarding performance and development is not directly available. Accordingly, current knowledge of how embryonic development and natural selection drive phenotypic evolution in vertebrates has been extended from studies performed at short temporal scales. We propose here the organization of the tetrapod body-axis as a model system to investigate the developmental origins of convergent evolution over hundreds of millions of years. The quantification of the primary developmental mechanisms driving body-axis organization (i.e. somitogenesis, homeotic effects and differential growth) can be inferred from vertebral counts, and recent techniques of three-dimensional computational biomechanics have the necessary potential to reveal organismal performance even in fossil forms. The combination of both approaches offers a novel and robust methodological framework to test competing hypotheses on the functional and developmental drivers of phenotypic evolution and evolutionary convergence.

## 1. Introduction

Charles Darwin described the process of evolution as a source of *endless forms* [1], but the skeletal resemblance among some biological designs (e.g. ichthyosaurs–dolphins–sharks or pterosaurs–bats–birds) demonstrates that some forms are more prone to evolve than others [2]. These morphologies evolve independently in many groups, over and over again and such convergent designs suggest limits on morphological diversity, but the factors responsible for those limits are not fully understood. A key approach to address this issue is to reveal the origins of convergent evolution.

Convergent evolution is a quasi-ubiquitous phenomenon but its origin has been largely debated in evolutionary theory [3,4]. Most surveys distinguish two different views for explaining convergence. The *externalist* view claims that convergent evolution is the result of the unfettered ability of natural selection to produce optimal solutions to repeated environmental problems, and the *internalist* view argues that convergence is the result of ‘constraints’ that hamper the production of phenotypic variants, hence leading to the more likely evolution of similar features [5–7]. The ‘adaptive’ view proposes that phenotypic variants of low fitness are produced during development but eliminated by selection, and the ‘constraint’ view proposes a limited number of variants due to rare (or impossible) developmental outcomes. More recent hypotheses predict that developmental constraints cause some morphologies to arise more frequently than others but natural selection discards those of the lowest fitness [8].

**Figure 1.**
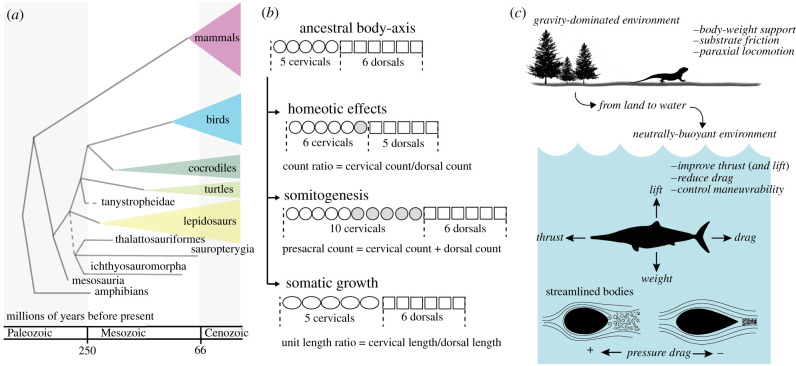
Body-axis macroevolution in marine tetrapods. (*a*) phylogenetic relationships of tetrapod lineages that include marine taxa, from [12]. (*b*) Primary developmental factors governing presacral axial organization [13]. (*c*) Changes in selective regimes from land-to-sea. Drag is minimized by streamlining the body (and appendages). Thrust and efficiency are increased by swimming strategies that use a lift-based oscillating hydrofoil [14].

Although both views present evidence that the diversity of Life is not *endless*, quantifying the strength of natural selection and developmental constraints in shaping convergent phenotypes over hundreds of millions of years is challenging because the information regarding performance and embryonic development is not directly available. As a consequence, although some studies have used an approach bridging palaeontology and developmental biology [9–11], our current knowledge of how developmental constraints and natural selection drive phenotypic evolution at geological timescales has been extrapolated from studies on laboratory organisms or from studies performed over short temporal scales. Here, we propose the organization of the tetrapod body-axis as a model system to decipher the relative contributions of developmental constraints and natural selection in shaping convergent body-plan configurations using the fossil record. We use the body-axis of tetrapods secondarily adapted to a marine lifestyle as a study case (figure 1*a*).

## 2. Developmental mechanisms of body-axis organization

The diversity of tetrapod body-axis proportions is enormous even for taxa adapted to the same physical environment. Surprisingly, this diversity results from the combination of three variables that are easily quantifiable in both recent [15] and fossil taxa [13,16], which are associated with the primary developmental mechanisms governing body-axis organization (figure 1*b*):

(i) The total number of vertebrae informs about the speed of the budding of the presomitic mesoderm in the formation of the new somites during the process of *somitogenesis* [17]. As the speed of new somite differentiation varies across lineages, somitogenesis is a compelling source of variation in body proportions across tetrapod phylogeny [18].
(ii) The relative numbers of vertebrae present in each region inform about changes in the *Hox gene* expression patterns [19–21] across species, which provide to somites their regional identities (i.e. cervical, thoracic, lumbar, sacral and caudal). The role for *Hox* genes in patterning axial skeletal regions has been demonstrated not only in mammals but also in other tetrapods [22–27], as well as in other vertebrates [28,29]. A major source of body-axis diversity among vertebrate clades is due to shifts in the expression boundaries of *Hox* genes (and other patterning genes) leading to variation in the distribution of vertebrae among morphological regions [18].

The axial skeletons of fishes are simply subdivided into trunk and tail regions [28,29], and the association of this regionalization with *Hox*-expression in Actinopterygii is well known [30]. Recent findings on the *Hox*-code expression and regionalization in the cartilaginous fish, *Leucoraja erinacea* [31] and in early ray-finned fish [32] predict an origin of *Hox*-based vertebral regionalization at the common ancestor of jawed vertebrates.

(iii) Finally, vertebral lengths inform about differential post-patterning growth of somites among axial regions, which determines the relative lengths of vertebrae within regions. Differential growth of somites among regions can result in evolutionary change in body proportions in the absence of other changes [13].

Therefore, the vertebral formula (number of vertebrae per region [33]) is a suitable proxy to investigate the main developmental mechanisms governing the macroevolution of axial column organization using fossils. During the evolution of amniotes, development can potentially respond to the same selective agent by at least three different pathways in the generation of adaptive body-plan configurations [13,33]. Marine reptiles had higher presacral numbers than their terrestrial close relatives, and this could be coupled with having long or short necks [16]. However, long necks can be acquired by altering somitogenesis (e.g. derived plesiosaurs), by homeotic changes (e.g. thalattosaurian crocodiles) or by increasing somatic growth (e.g. the archosauromorph reptile *Tanystropheus*) [16].

The response of the thoracic region of cetaceans and sirenians (all short-necked) to selective agents is similar to that of short-necked reptiles; the number of presacrals increases while retaining the ancestral cervical count. The axial system of pinnipeds and close terrestrial taxa is similar, as in placodont reptiles, suggesting that marine adaptations do not need to be coupled with changes in vertebral number [16].

Despite the low meristic variation of mammals [33], their vertebral formula may also vary. For example, while baleen whales have an increased body size retaining low vertebral counts, small oceanic dolphins possess a high number of short vertebrae [34].

## 3. The developmental potential and the tetrapod body-axis

**Figure 2.**
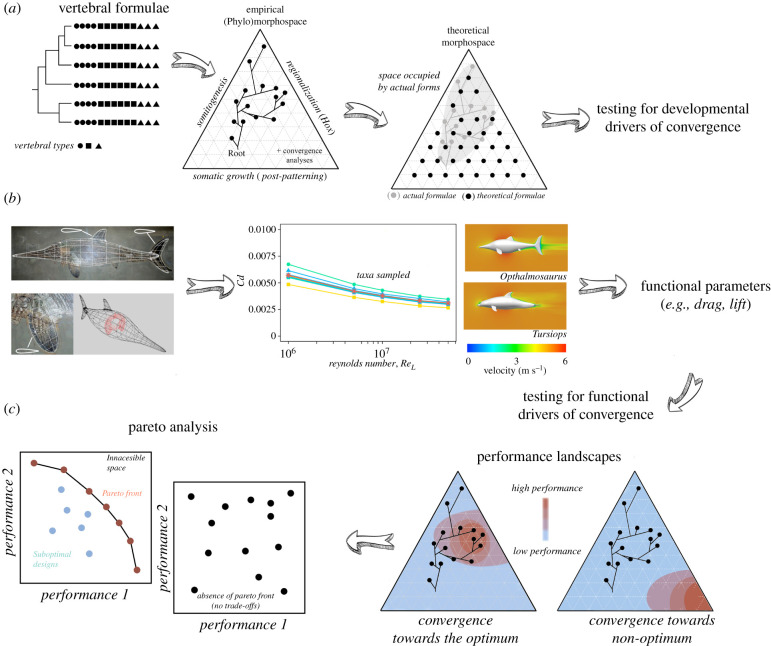
Schematic workflow (divided in three interconnected blocks) for testing competing hypotheses on developmental and functional triggers of evolutionary convergence in the tetrapod body-axis. (*a*) Derivation of empirical and theoretical (phylo)morphospaces from realized and unrealized combinations of the three developmental variables of body-axis organization to test for morphological convergence and to quantify how accessible design space is to developmental evolution; (*b*) the quantification of functional parameters derived from the application of biomechanical three-dimensional analyses (CFD) of realized designs, modified from [36]; (*c*) their integration into the morphospaces to develop performance landscapes to test for functional optimality of explored and unexplored regions of the morphospace, as well as for the existence of potential Pareto fronts. The later represents the set of designs that cannot be improved simultaneously in all tasks and allows for detecting performance trade-offs and it delimits the inaccessible design space from the region occupied by suboptimal designs.

The three variables that account for developmental changes in axial organization of the tetrapod axis could be analysed using theoretical morphology [35] to investigate the developmental potential [4]. An empirical morphospace depicted from actual combinations of the three developmental variables of body-axis organization (figure 2*a*) allows testing for developmental triggers of body-plan diversity at large-temporal scales. This can also manifest theoretical combinations of developmental variables with the potential to answer how much variation in the organization of the tetrapod body-axis has been explored by organic evolution (figure 2*a*). Different methods have been developed to study and quantify patterns of morphospace occupation [37–39] and phylomorphospaces [40] allowing to ascertain the evolutionary path of target lineages, including morphological convergence [41].

## 4. Adaptive performance in the tetrapod axis of living and extinct tetrapods

A direct consequence of body-axis organization is the shape of the external body, which has profound implications for locomotion [42]. Aquatic tetrapods have usually acquired efficient morphologies and propulsive mechanics for swimming, dictated by the need to increase speed, reduce drag, improve thrust output, enhance efficiency and control manoeuvrability in a neutrally buoyant environment [14] (figure 1*c*). Accordingly, they experienced profound morphological (and physiological) changes in their body plans, including the acquisition of streamlined bodies with fusiform shape, characterized by a rounded front and a tapered end [14]. This design effectively decreases the adverse pressure of the water column and minimizes flow separation, thereby reducing drag [43]. Streamlining of bodies and flattening of limb cross-sections are characteristics of the most specialized living aquatic tetrapods [14] because these changes minimize the energetic demands of swimming [44].

Computational fluid dynamics (CFD) is increasingly being used to investigate the relationship between body shape and locomotory performance in both living [45] and extinct [36,46,47] taxa. The results derived from CFD simulations allow the quantification of the forces exerted by the fluid on the three-dimensional model such as drag—forces opposing the relative motion of an object in fluid—or lift—forces acting perpendicular to motion direction, caused by flow deflection around the body or appendages. The derivation of dimensionless coefficients allows the comparison of the hydrodynamic efficiency among different models (e.g. lift-to-drag ratio).

CFD has been applied to infer swimming performance in extinct taxa such as in ichthyosaurs [36], plesiosaurs [42] and stem-gnathostomes [46]. For example, CFD has allowed to quantify the impact of body-plan evolution in ichthyosaurs on the energy demands of swimming [36]. This has revealed that ichthyosaurs produced low levels of drag for a given volume and their large bodies, as well as their efficient swimming modes, lowered the cost of steady swimming [36]. Troelsen *et al*. [47] used CFD to investigate the hydrodynamics of neck length and thickness in plesiosaurs, a group of Mesozoic marine reptiles with a unique body plan characterized by two pairs of flippers and an elongated neck. They [47] demonstrated that while neck elongation does not affect to drag during forward-swimming in plesiosaurs, thicker necks is a factor that substantially reduces drag.

Alternatively, analyses of osteological maximum range of motion (oROM) of virtually assembled vertebral columns in a specific pose [48,49] can be performed to quantify the degree of flexion and extension deployed by marine taxa which can reveal the swimming strategy deployed by extinct forms. Molnar *et al*. [48] investigated changes in oROM and intervertebral joint stiffness of thoracic and lumbar vertebrae with increasing aquatic adaptation through the evolution of crocodylomorphs. They concluded that joint stiffness in mediolateral flexion tended to decrease with adaptation to aquatic locomotion in thalattosuchians, but the trend seems to have reversed somewhat in other aquatic specialists [48].

## 5. Disentangling the developmental origins of convergent evolution in Deep Time

The combination of morphospaces obtained from the three developmental parameters that account for the diversity of the tetrapod axis (figure 2*a*) can be combined with those parameters that account for (hydrodynamic) efficiency—e.g. drag and lift obtained from CFD—(figure 2*b*) into performance landscapes [50] to quantify whether morphological convergence have occurred towards the functional optimum (figure 2*c*). Performance landscapes allow testing for functional drivers of morphological convergence quantifying the functional optimality of both occupied and unoccupied regions of the morphospace [51,52]. For example, Ferrón *et al*. [41] investigated the functional component of morphological convergence in the headshield of stem-gnathostomes using performance landscapes derived from CFD. They revealed similar hydrodynamic performances among species that converged towards the same regions of the morphospace, supporting that the evolution of similar morphologies in these groups may relate to functional drivers and the acquisition of similar ecologies.

Optimal designs are usually constrained by the existence of trade-offs between more than one task/function [53], impossible to infer from adaptive landscapes. To this regard, Pareto efficiency theory (PET) can assess optimality between two traits or more [54]. Tendler *et al*. [55] used PET to quantify shape variation in ammonoid shells and concluded that they fall within a square pyramidal region of the morphospace whose vertices correspond to five putative tasks, including hydrodynamic efficiency, shell economy, compactness and rapid shell growth [55]. Moreover, the distance from each species to each vertex, indicates the relative importance of each task to the lifestyle of that species. The investigation of how these patterns of morphospace occupation changed across different mass extinctions led Tendler *et al*. [55] to investigate ammonoids' ecological responses to these catastrophic events.

The combination of adaptive landscape evaluation and PET (figure 2*c*) can be used to explicitly test whether evolution has explored all functional optimal morphologies, whether many species are functionally suboptimal, whether unrealized morphologies are functionally poor and whether some optimal morphologies have never been achieved in evolutionary history.

## 6. Future directions

The developmental mechanisms governing the tetrapod axis have been already studied in living and fossil taxa [13,16,34] and three-dimensional functional analyses (e.g. CFD and oROM) have been applied for inferring swimming performance in extinct marine vertebrates [36,46,47]. We emphasize that the combination of both types of analyses for the study of vertebrate body-axis using our proposed approach (figure 2) could be a new avenue for future research to disentangle the developmental origins of convergent evolution in Deep Time.

Our proposed approach could be of potential application to decipher the relative contributions of development and adaptation in the generation of convergent body-axis in a wide variety of taxa beyond marine tetrapods. For example, this could be the case of putatively convergent tetrapods towards an airborne locomotion such as birds, pterosaurs and bats or secondarily adapted species of mammals and reptiles towards specific locomotory demands (e.g. arboreality, cursoriality, etc.). Beyond tetrapods, the investigation of developmental changes in the body-axis of fish as a response of different aquatic adaptations (*sensu* [28]) could be also a potential avenue for future research.

Altogether, this approach offers a novel methodological framework to test competing hypotheses about the functional and developmental drivers of phenotypic evolution and morphological convergence, using the body-axis as a model system.

## Data Availability

This article has no additional data.
